# Sleep restriction can attenuate prioritization benefits on declarative memory consolidation

**DOI:** 10.1111/jsr.12424

**Published:** 2016-06-13

**Authors:** June C. Lo, Kelly A. Bennion, Michael W. L. Chee

**Affiliations:** ^1^ Centre for Cognitive NeuroscienceNeuroscience and Behavioral Disorders ProgramDuke‐NUS Medical SchoolSingapore; ^2^ Boston CollegeChestnut HillMAUSA

**Keywords:** adolescents, memory, preferential consolidation, reward, sleep, sleep loss

## Abstract

As chronic sleep restriction is a widespread problem among adolescents, the present study investigated the effects of a 1‐week sleep restriction (SR) versus control period on the consolidation of long‐term memory for prose passages. We also determined whether the benefit of prioritization on memory is modulated by adequate sleep occurring during consolidation. Fifty‐six healthy adolescents (25 male, aged 15–19 years) were instructed to remember a prose passage in which half of the content was highlighted (prioritized), and were told that they would receive an additional bonus for remembering highlighted content. Following an initial free recall test, participants underwent a 7‐night period in which they received either a 5‐h (SR) or 9‐h (control) nightly sleep opportunity, monitored by polysomnography on selected nights. Free recall of the passage was tested at the end of the sleep manipulation period (1 week after encoding), and again 6 weeks after encoding. Recall of highlighted content was superior to that of non‐highlighted content at all three time‐points (initial, 1 week, 6 weeks). This beneficial effect of prioritization on memory was stronger 1 week relative to a few minutes after encoding for the control, but not the SR group. N3 duration was similar in the control and SR groups. Overall, the present study shows that the benefits of prioritization on memory are enhanced over time, requiring time and sleep to unfold fully. Partial sleep deprivation (i.e. 5‐h nocturnal sleep opportunity) may attenuate such benefits, but this may be offset by preservation of N3 sleep duration.

## Introduction

Prioritization is helpful in keeping the volume of information we encounter each day to a manageable level. This may include presenting information in bold or highlighted text (Lorch, 1989), explicitly telling individuals to remember information (Hulstijn, 2008), and/or offering rewards for later memory (Fischer and Born, 2009). While these strategies are used commonly, it is unclear how their benefit evolves and for how long these enhancing effects persist. Of specific interest to the present work is whether sleep modulates the retrieval of these prioritized versus non‐prioritized memoranda over time.

A wealth of literature has shown that manipulating encoding instructions affects subsequent memory. For example, memory performance is typically better if, during encoding, individuals are aware that the information will be tested later (i.e. intentional encoding) than if they are not (i.e. incidental encoding; Hulstijn, 2008). Even within intentional encoding conditions, prioritizing certain information by associating it with reward enhances future retrieval. For instance, cues signalling subsequent reward are better remembered than neutral cues (Wittmann *et al*., 2005), as are cues signalling high‐value relative to low‐value reward (Adcock *et al*., 2006). At least one study suggests that these reward effects may be long‐lasting, demonstrating an enhancing effect of reward on motor memory that arose by 6 h after training, was strengthened 24 h after training and persisted 30 days after training (Abe *et al*., 2011). It is not known if reward has similar lasting effects on declarative memory, and whether reward affects memory for stimuli similar to those encountered in educational or vocational settings (e.g. prose passages).

Recent work suggests that the benefits of prioritization on memory take time to develop. For example, reward anticipation during encoding leads to greater memory benefits after a delay relative to immediately after encoding. Specifically, studies have shown that associating a reward cue with neutral images resulted in enhanced memory for those images following a 1‐week delay, but not immediately (Murayama and Kitagami, 2014), and that recognition was enhanced for high‐reward relative to low‐reward images after a 24‐h delay, but not immediately after encoding (Spaniol *et al*., 2013). In both cases, a period of sleep separated learning and test, which could have resulted in selective strengthening of rewarded information. Previous literature on the effects of sleep on memory indicates that there is a benefit for memories ‘tagged’ during or soon after encoding (Stickgold and Walker, 2013). While several factors can lead to stimuli being tagged as important to remember (Oudiette and Paller, 2013), of particular relevance to the present study is work showing that the sleep‐dependent gain in motor performance is greater for sequences associated with a monetary reward relative to those that are not (Fischer and Born, 2009). Similarly, simply the expectancy of a memory test can lead to sleep‐dependent gains in declarative, procedural, and visuospatial memory (Van Dongen *et al*., 2012; Wilhelm *et al*., 2011).

In elucidating the conditions under which these tagging benefits may occur, it should be noted that previous studies have included adult participants who either had an adequate amount of overnight sleep (Bennion *et al*., 2014, 2015a; Cunningham *et al*., 2014) or a daytime nap (Bennion *et al*., 2016; Oudiette *et al*., 2013) during the consolidation interval. Partial or total sleep deprivation may attenuate these tagging benefits. Given the high prevalence of chronic sleep restriction in teenagers (National Sleep Foundation, 2006) (i.e. less than the optimal 8–10 h of nightly sleep; Hirshkowitz *et al*., 2015), investigating tagging benefits on declarative memory within this adolescent sample is novel and critical to understanding this phenomenon in a real‐life setting.

The present study utilized a 7‐night sleep manipulation (5‐ versus 9‐h nocturnal sleep opportunity) within a sample of 15–19‐year‐old students to elucidate how the effects of prioritization on memory for a prose passage may be driven by sufficient sleep occurring during consolidation. Particularly, we addressed three questions: Does prioritizing information by rewarding subsequent recollection of highlighted (versus non‐highlighted) content lead to enhanced memory for the highlighted content? If so, how long do these beneficial effects persist and are they still observed weeks after encoding? Lastly, to what extent do such benefits of prioritization on memory depend on adequate sleep during consolidation?

## Methods

### Participants

Sixty participants between ages 15 and 19 years were invited to participate in a 2‐week protocol, as well as for a follow‐up recall test 6 weeks after encoding. Reported here are data from the 56 participants [25 males, mean ± standard deviation (SD) age: 16.6 ± 1.1 years] who complied with experimental procedures and completed the 2‐week protocol, as well as the 45 participants (19 males, 16.6 ± 1.1 years) who returned for the follow‐up visit. All participants had a body mass index (BMI) < 30. They had no medical, psychiatric, or sleep disorders, showed no signs of habitual short sleep, consumed fewer than five cups of caffeinated beverages a day, and did not travel across more than two time zones during the month prior to the experiment. The study was approved by the Institutional Review Board of the National University of Singapore, and written informed consent was obtained from each participant and a legal guardian. A detailed description of recruitment and screening procedures may be found in Lo *et al*. (2016) and Supporting information, Appendix S1.

Participants were randomized into either the sleep restriction (SR) or control groups; they were not informed about the grouping until the first day of the 2‐week protocol. The SR (*n* = 30) and control (*n* = 26) groups did not differ in age, gender distribution, BMI, consumption of caffeinated beverages, sleep efficiency, time in bed, or total sleep time on weekdays or weekends (all *P*s > 0.22), as measured by actigraphy (Actiwatch AW‐2, Respironics, Inc., Murraysville, PA, USA) around their non‐dominant wrist for 1 week during screening (Table 1).

**Table 1:** Participant characteristics of the control and sleep restriction (SR) groups

	Control group		SR group		*t*	*P*
	Mean	SD	Mean	SD		
*n*	26	–	30	–	–	NS
Age (years)	16.81	1.17	16.43	0.94	1.33	NS
Gender (% males)	42.30	–	46.70	–	0.11	NS
Body mass index	20.38	2.55	20.43	2.88	0.07	NS
Caffeinated drinks per day	0.54	0.79	0.75	0.55	1.18	NS
TIB on weekdays (h)	6.09	0.85	6.40	0.94	1.24	NS
TIB on weekends (h)	8.45	1.25	8.46	1.08	0.99	NS
TST on weekdays (h)	5.37	0.73	5.61	0.86	1.11	NS
TST on weekends (h)	7.53	1.14	7.46	1.10	0.21	NS
Sleep efficiency (%)	88.45	4.66	87.86	5.46	0.42	NS

Sleep data were measured by wrist‐worn actigraphy for 1 week during screening and scored with Actiware software (version 6.0.2). SD, standard deviation; TIB, time in bed; TST, total sleep time; SD, standard deviation; NS, not significant.

### Two‐week protocol and follow‐up visit

One week prior to the study, participants were required to adhere to a specified sleep–wake schedule that provided a 9‐h nocturnal sleep opportunity (23:00–08:00 hours). This was verified using an Actiwatch and was intended to minimize any effect of previous sleep restriction on cognitive performance.

**Figure 1:**
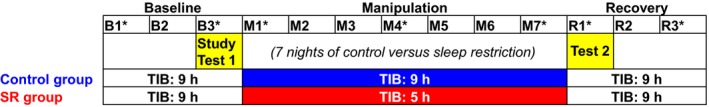
This protocol started with three baseline nights [B1–B3; 9 h time in bed (TIB)], followed by seven nights of sleep manipulation (M1–M7) with 9 h TIB for the control group and 5 h TIB for the sleep restriction (SR) group. Both groups had 9 h TIB during recovery nights (R1–R3). Encoding (study) took place on the evening of the third baseline night. Retrieval took place before and after the sleep manipulation period (test 1, test 2) and 6 weeks after encoding (test 3; not depicted). Asterisks indicate nights that participants’ sleep was monitored by polysomnography. Sleep–wake patterns were monitored continuously by actigraphy except during night B1, when all Actiwatches were charged.

This 2‐week protocol was conducted in a boarding school; see Lo *et al*. (2016) or Supporting information, Appendix S1 for a description of participants’ accommodations and activities. During the first 3 nights (B1–B3), all participants had a 9‐h nocturnal sleep opportunity (23:00–08:00 hours). This was followed by a 7‐night manipulation period (M1–M7) in which the SR group had 5‐h (01:00–06:00 hours) and the control group had 9‐h (23:00–08:00 hours) sleep opportunities. The protocol ended with three nights of 9‐h recovery sleep (R1–R3: 23:00–08:00 hours) for both groups (Fig. 1).

Polysomnographic (PSG) recordings were obtained on seven nights: B1 and B3 for adaptation and baseline assessment, M1, M4, and M7 to monitor sleep changes from the beginning to end of the manipulation period, and R1 and R3 for characterizing recovery sleep. PSG was recorded with a SOMNOtouch^TM^ RESP (SOMNOmedics, Randersacker, Germany) system that included electro‐oculography (EOG), electromyography (EMG), and electroencephalography (EEG) leads (C3 and C4 in the international 10–20 system), with each electrode referenced to the contralateral mastoid. The ground and reference electrodes were placed at Cz and FPz. Impedance was kept below 5 kΩ for EEG electrodes and below 10 kΩ for EOG and EMG electrodes. Signals were sampled at 256 Hz and filtered between 0.2 and 35 Hz for EEG and EOG. Sleep data were scored using the FASST toolbox (http://www.montefioreulg.ac.be/~phillips/FASST.html), with EEG signals band‐pass filtered between 0.2 and 25 Hz. Scoring was performed by trained technicians according to the standards of the American Academy of Sleep Medicine (Iber *et al*., 2007).

Six weeks after encoding, participants were invited for a follow‐up session under the expectation of being called back for a social event. Upon return, they received an unexpected memory test, assessing their delayed retention of the passage.

### Prose passage

Participants were instructed to learn a prose passage (Sullivan, 2005; Supporting information, Appendix S2). The passage had four sentences, altogether containing 25 idea units. A prose passage was used, rather than other stimuli such as word lists, to approximate the experience of studying as relevant to educational domains. Prioritization of certain content was achieved by highlighting two sentences (13 idea units) in yellow. Participants were informed at encoding that bonus points would be offered for successful retrieval of the highlighted (HL) content and that the best performer would be rewarded.

Encoding occurred 2 h prior to bedtime (at 21:00 hours) on the third baseline night (B3; Fig. 1), in which participants were instructed that their memory for the passage would be tested later. Retrieval was tested at three separate time‐points: approximately 10 min after encoding (initial), after the 1‐week manipulation period, and 6 weeks after encoding. Each retrieval session consisted of a free recall test: Participants were asked to write down any content from the passage that they could remember, with the measure of performance being the percentage of correctly recalled idea units.

Between encoding and the initial test (approximately 10 min later), participants were engaged in another task, so it is unlikely that they rehearsed the information. At 1 and 6 weeks after encoding, participants did not know that their memory for the passage would be tested, and as such, it is unlikely that they used any strategies (e.g. writing down content) to remember the information intentionally.

### Statistical analyses

All statistical analyses were performed with SPSS version 22.0 (IBM, Chicago, IL, USA). We ran a repeated‐measures analysis of variance (ANOVA) (reporting effect size as *ηp* ^*2*^; small: 0.1, medium: 0.3, large: 0.5) to determine whether memory differed as a function of delay length and prioritization, and between groups. Statistical significance of pairwise contrasts was examined with *post‐hoc *independent‐ or paired‐sample *t*‐tests (reporting effect size as Cohen's *d*; small: 0.2, medium: 0.5, large: 0.8).

Polysomnography analyses, comparing the duration of each sleep stage between the control and SR groups during the baseline and manipulation periods, were conducted using independent‐sample *t‐*tests. We used Pearson's correlations to investigate whether there were any linear associations between sleep macrostructure (i.e. time spent in each sleep stage during the 1‐week manipulation period, here averaged across manipulation nights M1, M4, and M7) and memory performance.

## Results

### Effects of prioritization and delay length on memory

**Figure 2:**
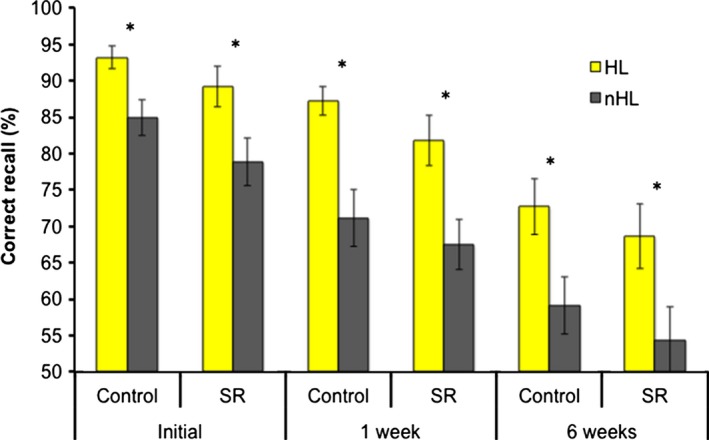
Free recall performance of the control and sleep restriction (SR) groups. This bar graph shows the mean percentage of highlighted (HL) and non‐highlighted (nHL) idea units that were recalled correctly a few minutes after learning (initial), after the sleep opportunity manipulation period (1 week), and 6 weeks after encoding (6 weeks). Error bars reflect standard error of the mean.

There was a significant main effect of prioritization on recall performance (*F*_(1,43)_ = 45.14, *P *< 0.001, *ηp* ^*2*^ *= *0.51), with consistently better recall of prioritized (highlighted) information than non‐prioritized information across all time points (initial: *t*_(55)_ = 4.99, *P *< 0.001, *d = *1.35; 1 week after encoding: *t*_(55)_ = 6.28, *P* < 0.001, *d = *1.69; 6 weeks after encoding: *t*_(44)_ = 5.56, *P* < 0.001, *d = *1.68; Fig. 2). The effect of delay length was statistically significant (*F*_(2,42)_ = 55.96, *P < *0.001, *ηp* ^*2*^ *= *0.73), indicating forgetting over time. *Post‐hoc* paired‐sample *t‐*tests determined that significant forgetting occurred from the initial recall test to after the 1‐week manipulation period (*t*_(55)_ = 7.98, *P* < 0.001, *d = *2.15) and from after the manipulation period to the follow‐up visit 6 weeks after encoding (*t*_(44)_ = 8.53, *P* < 0.001, *d = *2.57; Fig. 2). There was no main effect of group (*F*_(1,43)_ = 1.13, *P = *0.29, *ηp* ^*2*^ *= *0.026), nor was there an interaction between group and prioritization (*F*_(1,43)_ = 0091, *P = *0.76, *ηp* ^*2*^ *= *0.002) or group and delay length (*F*_(1,43)_ = 0.005, *P = *0.995, *ηp* ^*2*^ *< *0.001).

### Interaction of prioritization and delay length on memory: importance of sleep during consolidation

The repeated‐measures ANOVA showed a significant interaction of prioritization and delay length on memory performance (*F*_(2,42)_ = 3.61, *P = *0.036, *ηp* ^*2*^ *= *0.15), suggesting that non‐highlighted (nHL) content was forgotten faster than HL content; Fig. 2. We then confirmed this finding with another analysis, using a measure of ‘prioritization benefit’ (defined as memory for HL content minus memory for nHL content). Specifically, *post‐hoc* paired‐sample *t‐*tests showed that the benefit of prioritization on memory was greater after the 1‐week manipulation period relative to initially (*t*_(55)_ = 2.77, *P = *0.008, *d = *0.75).^1^ The prioritization benefit on memory did not continue to increase beyond 1 week, but remained stable: The prioritization benefit 6 weeks after encoding was equivalent to that after the 1‐week manipulation period (*t*_(44)_ = 0.40, *P = *0.69, *d = *0.069).

**Figure 3:**
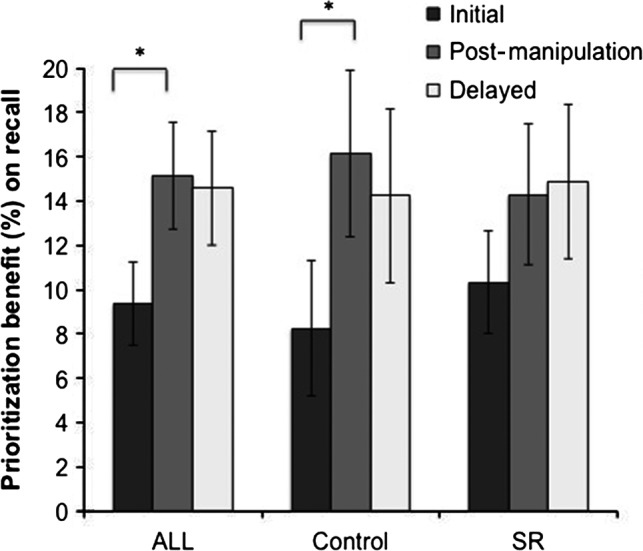
Prioritization benefit on memory. For all participants [control and sleep restriction (SR) groups combined] and for the control group alone, the benefit of prioritization (percentage of highlighted idea units correctly recalled minus that of non‐highlighted idea units) on memory was strengthened after the manipulation period relative to minutes after encoding. However, this did not hold true for the SR group. Error bars reflect standard error of the mean.

Further analyses on the measure of prioritization benefit showed that its strengthening after a 1‐week delay relative to initially may be driven by adequate sleep (i.e. a 9‐h nocturnal sleep opportunity) during the manipulation period. Although the three‐way interaction (prioritization × delay length × group) from the ANOVA was not significant (*F*_(2,42)_ = 0.90, *P = *0.41, *ηp* ^*2*^ *= *0.041), the benefit of prioritization on memory was greater 1 week after encoding relative to initially only for the control group (*t*_(25) _= 2.47, *P = *0.021, *d = *0.99), and not the SR group (*t*_(29)_ = 1.45, *P = *0.16, *d = *0.26; Fig. 3).

### Polysomnography results: Group differences

**Figure 4:**
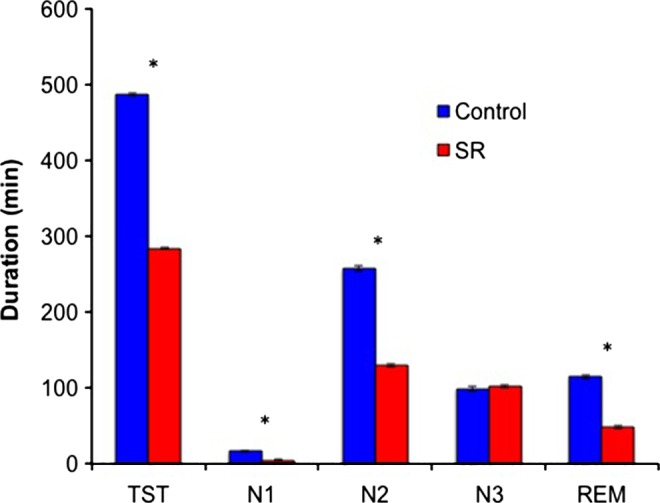
Sleep characteristics, separated by group [control, sleep restriction (SR)], averaged across manipulation nights 1, 4, and 7. Error bars reflect standard error of the mean.

Polysomnography data on the baseline night revealed no significant differences between groups in total sleep time (TST), wake after sleep onset (WASO), sleep onset latency and the duration of N1, N2, N3, and rapid eye movement (REM) sleep (all *P*s > 0.12). Conversely, on all manipulation nights (M1, M4, M7), relative to the control group, participants in the SR group had shorter TST, WASO, sleep latency, N1, N2, and REM sleep (all *P*s < 0.001; Fig. 4; Supporting information, Appendix S3); the only sleep parameter that did not differ between groups was the duration of N3 (M1: *P = *0.23; M4: *P = *0.36; M7: *P = *0.10).

**Figure 5:**
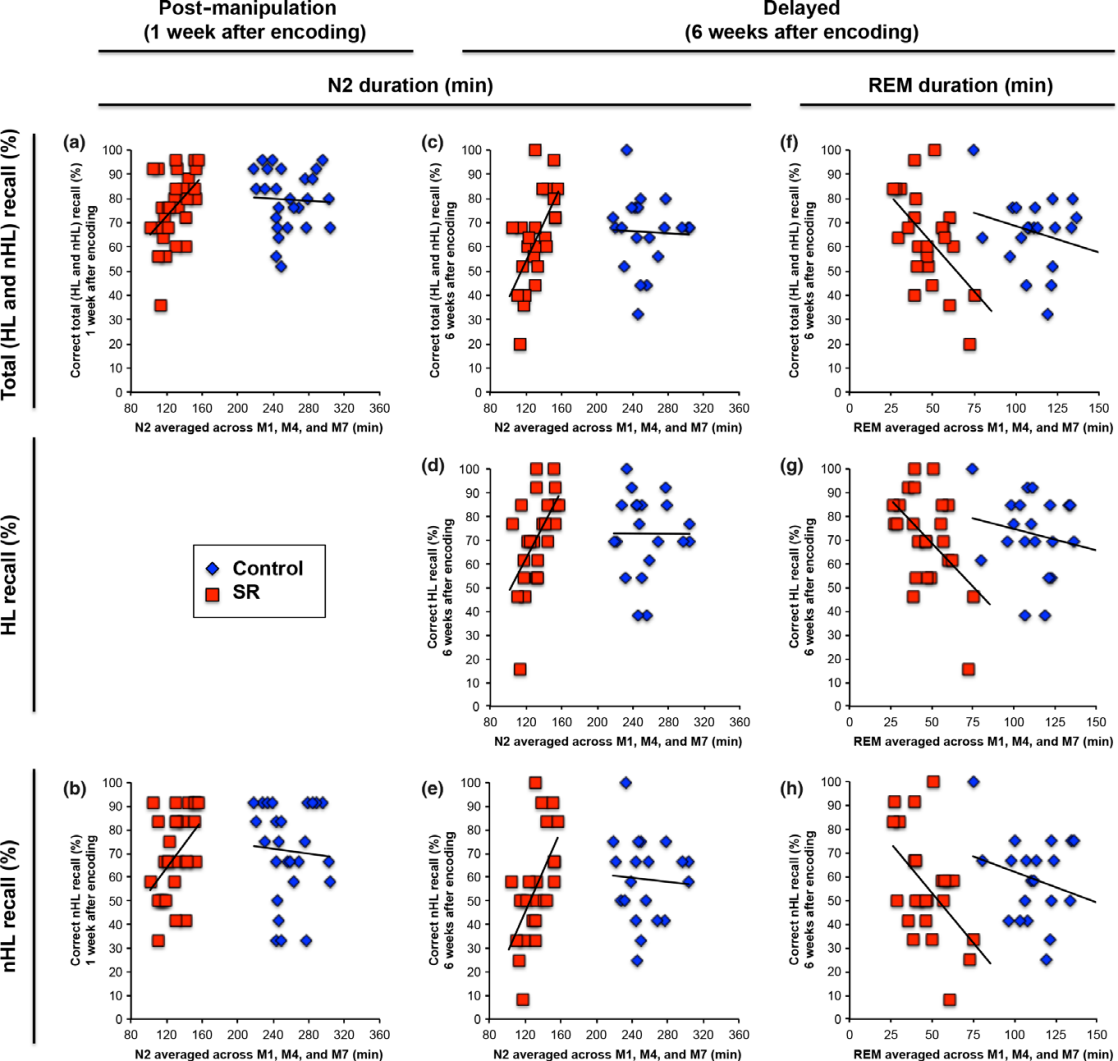
Relation between sleep and memory performance at the post‐manipulation (a–b) and delayed (c–h) time points. Regression lines indicate the linear associations of the duration (averaged across manipulation nights M1, M4, and M7) of N2 (a–e) and rapid eye movement (REM) sleep (f–h) with memory for total content [i.e. highlighted (HL) and non‐highlighted (nHL) content; a,c,f], HL content (d,g), and nHL content (b,e,h). Data for the control and sleep restriction (SR) groups are illustrated in blue diamonds and red squares, respectively. All graphs depict significant correlations between sleep and memory for the SR, but not control, groups.

In the control group, there was no relation between sleep macrostructure and any aspect of memory performance. In the SR group, longer average N2 duration was associated with better memory for total (i.e. HL and nHL) content and nHL content at the post‐manipulation time point (both *r* = 0.45, *P* = 0.014; Fig. 5a‐b; Table 2), and better memory for total, HL, and nHL content at the delayed time point (*r *> 0.56, *P* < 0.005; Fig. 5c–e). Also in the SR group, there was an inverse linear relation between average REM duration and memory for total, HL, and nHL (*r *> −0.50, *P* < 0.013) content at the delayed time point (Fig. 5f–h). Fisher's *Z*‐tests showed that the only statistically significant differences between groups of the correlation coefficients between sleep macrostructure and memory performance were between average N2 duration and memory for total and nHL content at the delayed time point (*Z *> 2.22, two‐tailed *P* < 0.026; Table 2).

**Table 2:** Correlations of memory performance at the post‐manipulation and delayed time points with the durations of TST, N1, N2, N3 and REM sleep, averaged across manipulation nights M1, M4, and M7

	Post‐manipulation						Delayed					
	Control		SR		Fisher's *Z*		Control		SR		Fisher's *Z*	
	*r*	*P*	*r*	*P*	*Z*	*P*	*r*	*P*	*r*	*P*	*Z*	*P*
TST												
Total	0.05	0.83	−0.12	0.52	0.58	0.56	0.04	0.88	−0.24	0.25	0.86	0.39
HL	0.14	0.50	−0.12	0.55	10.43	0.15	−0.04	0.86	−0.28	0.19	0.75	0.45
nHL	−0.02	0.93	−0.10	0.61	0.29	0.77	0.11	0.65	−0.18	0.41	0.90	0.37
N1												
Total	0.15	0.48	0.01	0.96	0.49	0.62	0.04	0.86	−0.02	0.92	0.20	0.84
HL	0.14	0.49	0.05	0.81	10.38	0.17	0.20	0.39	0.04	0.85	0.50	0.62
nHL	0.11	0.60	−0.03	0.90	0.47	0.64	−0.13	0.57	−0.08	0.72	0.16	0.87
N2												
Total	−0.05	0.83	**0.45**	**0.01**	10.85	0.06	−0.04	0.88	**0.62**	**0.00**	**20.34**	**0.02**
HL	0.01	0.96	0.33	0.08	10.16	0.25	−0.00	0.99	**0.56**	**0.01**	10.95	0.05
nHL	−0.07	0.75	**0.45**	**0.01**	10.93	0.06	−0.06	0.80	**0.58**	**0.00**	**20.22**	**0.03**
N3												
Total	0.02	0.91	−0.28	0.14	0.78	0.44	0.20	0.39	−0.18	0.40	10.13	0.26
HL	−0.10	0.62	−0.14	0.46	0.40	0.69	0.03	0.89	−0.18	0.40	10.16	0.25
nHL	0.09	0.68	−0.34	0.07	0.18	0.86	0.33	0.16	−0.16	0.47	0.94	0.35
REM												
Total	0.02	0.94	−0.23	0.23	0.87	0.38	−0.24	0.31	−**0.55**	**0.01**	10.15	0.25
HL	0.20	0.32	−0.25	0.19	10.60	0.11	−0.17	0.47	−**0.50**	**0.01**	10.16	0.25
nHL	−0.09	0.65	−0.15	0.44	0.20	0.84	−0.24	0.31	−**0.50**	**0.01**	0.93	0.35

REM, rapid eye movement; SR, sleep restriction; HL, highlighted; nHL, non‐highlighted.

Post‐manipulation = 1 week after encoding; delayed = 6 weeks after encoding.

M1, M4, and M7 represent the first, fourth and seventh sleep opportunity manipulation nights, respectively.

Bolded values are significant at p < .05.

## Discussion

In this study, we investigated whether prioritization influences memory recall at multiple time points of the retention interval, and whether sleep duration during consolidation modulates these prioritization effects. Our findings suggest that the effects of prioritization are robust and long‐lasting, present initially, 1 week, and 6 weeks after encoding. Secondly, these benefits of prioritization evolve over time, increasing from initial recall to 1 week after encoding, but stabilizing afterwards up to 6 weeks after encoding. Lastly, this strengthening of the prioritization benefit over time may be driven by sufficient sleep occurring during the consolidation interval (i.e. 7 nights of a 9‐ versus 5‐h nocturnal sleep opportunity); only in the control group was the prioritization benefit on memory enhanced 1 week after encoding relative to at the initial assessment.

### Prioritization has long‐lasting benefits on memory for prose passages

Prioritization can enhance memory in the form of emotional salience (Hamann, 2001), evolutionary significance (Nairne *et al*., 2008), or reward (Castel *et al*., 2007), as in the present study. Here, we show that beneficial effects of reward on memory, as indicated by better memory for HL versus nHL content, are present a few minutes, 1 week, and even 6 weeks after encoding. These findings are consistent with several studies documenting the benefit of reward on declarative memory consolidation (e.g. Eysenck and Eysenck, 1982), and also extend this literature by showing that such effects persist over a much longer delay than studied previously. The prioritized content in the present study may also benefit from effects of typographical cueing (i.e. highlighting), which has been shown to enhance memory for cued content without affecting memory for uncued content (Lorch, 1989).

### The benefits of prioritization on memory are strengthened over time

The benefits of prioritization on memory were stronger after 1 week but non‐significantly stronger (*P* = 0.10) after 6 weeks. Even though a prioritization benefit was present minutes after encoding, the temporal evolution of the prioritization effect suggests that the beneficial effects of prioritization on memory occur during consolidation processes (Hamann, 2001; McGaugh, 2000), rather than encoding processes. While the latter could result from a preferential allocation of study time (Castel *et al*., 2002) to HL versus nHL content, our results are consistent with an account of dopaminergic modulation of hippocampal‐based consolidation, with dopamine likely affecting hippocampal plasticity and memory during the hours after encoding (Shohamy and Adcock, 2010), and especially during sleep (Perogamvros and Schwartz, 2012). This could explain why previous studies have found that reward cues led to increased memory following 24‐h (Spaniol *et al*., 2013) and 1‐week (Murayama and Kitagami, 2014) delays, but not immediately.

The strengthening of the prioritization benefit during the 1‐week manipulation period could be due not only to consolidation, but also pruning or weakening of non‐prioritized memories (Maquet, 2001). Particularly, the concept of synaptic down‐scaling states that synapses that are activated strongly (i.e. those corresponding to prioritized content in the present study) are relatively preserved, whereas those less activated are downscaled and eventually forgotten (Nere *et al*., 2013; Tononi and Cirelli, 2014); indeed, there was faster forgetting of nHL relative to HL content (Fig. 2). By this account, we suggest that during the 1‐week manipulation period, important memories were more likely to be consolidated during sleep and subsequently remembered, while less important memories were more likely to be weakened and forgotten—together reflecting a complex and adaptive modulation of memories over time.

### The impact of sleep in retaining important memories

Although it is well known that sleep facilitates and, conversely, that sleep loss impairs declarative memory consolidation (Walker, 2008), we found no significant differences in memory performance (at any time point, for HL and nHL content) between the SR and control groups. While this might seem surprising, our results are consistent with a similar study by Voderholzer *et al*. (2011), who showed that sleep restriction (with adolescents obtaining as little as 5 h of nocturnal sleep) during four consecutive nights did not impact declarative or procedural memory consolidation significantly. It was suggested that preservation of N3 duration in the sleep‐restricted conditions protected these participants from memory impairment. Congruent with this previous finding, the SR group in the present study maintained their absolute amount of N3 sleep during the manipulation period despite a marked reduction in TST and all other sleep stages. This preservation of N3 sleep is reassuring, given that the majority of adolescents, and especially those in East Asia, sleep less than the recommended 8–10 h a night (Hirshkowitz *et al*., 2015). Together, these findings are consistent with previous literature showing that N3 promotes declarative memory consolidation (Plihal and Born, 1997), and suggest that N3 may offset detrimental effects of sleep restriction on memory.

Results suggest that under conditions of sleep restriction, N2 sleep duration may contribute to declarative memory consolidation. While N2 sleep duration was markedly reduced in the SR relative to the control group, it nonetheless predicted memory for nHL and total content in the SR group 1 week after encoding, and HL, nHL, and total content 6 weeks after encoding. This is in contrast to previous studies that have shown no relation between N2 sleep duration and memory performance following a full night of sleep (Schabus *et al*., 2004) or afternoon nap (Ruch *et al*., 2012), although it should be noted that only at the delayed time point were any differences in correlation coefficients between groups statistically significant. Unexpectedly, under conditions of sleep restriction, greater REM sleep was associated with poorer memory after an extended time interval (here, 6 weeks). Future research should continue to investigate how REM sleep obtained during sleep restriction affects memory after various delay lengths, particularly to elucidate contexts under which a negative association is found. We reiterate that most studies seeking to establish the contribution of sleep architecture to memory allow sleep for an optimal duration during post‐learning nights and that the present findings occurring in the context of sleep restriction should be interpreted cautiously and with the caveat of multiple nights of sleep restriction in mind.

Our results suggest that memory in both the SR and control groups benefited from an interaction between encoding‐ and consolidation‐based processes, whereby the association of highlighted content with anticipated reward tagged these statements as important to remember during encoding, leading to their preferential reactivation and consolidation during sleep (e.g. Bennion *et al*., 2015b). This concept is reminiscent of synaptic tagging (Frey and Morris, 1997), although with a key difference. In classic accounts of synaptic tagging, and in recent studies in which participants were informed retroactively that certain stimuli had future relevance (Fischer and Born, 2009), the importance of the tagged memoranda was disclosed after encoding. In the present study, ‘tagging’ occurred during encoding; participants were aware during the study that they would receive a bonus for subsequent recall of prioritized information. Comparable performance between the SR and control groups suggests that in this sample of adolescents, ‘tagged’ information was consolidated preferentially even under conditions of suboptimal sleep—an effect that again may be driven by a preservation of N3 sleep. However, as only the control group showed a benefit of highlighting on memory that was stronger after 1 week relative to initially, these data suggest that restricting sleep during consolidation attenuates these tagging benefits.

### Limitations and future directions

We view this study as an important step in better understanding how prioritization affects long‐term memory consolidation, and how prioritization benefits are modulated by sleep obtained during consolidation. However, there are limitations that could be addressed in future work. First, testing memory after a 24‐h delay in addition to time points assessed here (minutes, 1 week, 6 weeks after encoding) would elucidate the time course of prioritization benefits on memory. Specifically, it would clarify if the prioritization benefits that were stronger 1 week relative to minutes after encoding require multiple nights to evolve, or perhaps only 1 night of sleep. Secondly, while memory here was assessed with free recall of a prose passage, future work would benefit from the use of other stimuli (e.g. word lists, images) to determine whether the benefits of prioritization, and the effect of sleep on such benefits, can be generalized to other stimuli. Lastly, while logistics and the sheer cost of running a 2‐week overnight study may preclude this, using a within‐subjects (versus between‐subjects) design could provide stronger evidence that sleep restriction may attenuate tagging benefits on consolidation.

## Conclusion

Prioritizing information leads to beneficial effects on memory that are stronger 1 week relative to minutes after encoding, and still present after 6 weeks. This enhancement in preferential consolidation of memory for prioritized over non‐prioritized content may not occur if sleep is restricted during the consolidation period. However, relative to prioritization, the benefit of sleep on delayed recall, even at 1 week, is smaller than what one might expect from the literature advocating the importance of sleep on memory consolidation (Diekelmann and Born, 2010). The preservation of N3 sleep despite sleep restriction may be contributory, and speaks to the resilience of adolescents in the context of declarative memory consolidation.

## Conflict of interest

The authors declare no conflicts of interest.

## Author contributions

JCL and MWLC developed the study concept and design. Data were collected by JCL. KAB performed the data analysis and drafted the manuscript. JCL and MWLC provided critical revisions. All authors approved the final version of the manuscript for submission.

## Supporting information


**Appendix S1**. Information on participants' recruitment, screening, accommodations and daily activities during the 2‐week experimental protocol. For additional information, see Lo and colleagues (2016).

**Appendix S2**. Prose passage read by participants, including instructions in bold type. The prioritization of content was manipulated by including highlighted and non‐highlighted statements, with participants told that they would receive an additional reward for subsequent memory of highlighted information. The vertical lines (shown here, but not to participants) depict the separation of idea units.

**Appendix S3.** Sleep stage duration in minutes (mean ± standard error), separated by group (control, SR) and manipulation night (M1, M4, M7).

## Footnotes

1 Note that while the *post‐hoc* analysis on the prioritization benefit includes 56 participants and the ANOVA reporting the significant prioritization by delay length interaction includes 45 participants (i.e. participants who were tested at all three time points, including the follow‐up visit 6 weeks after encoding), a subsequent ANOVA run on all 56 participants assessing memory for the first two time points showed that the prioritization by delay length interaction was nonetheless significant (*F*_(1,54)_ = 8.01, *P = *0.007, *ηp* ^*2*^ *= *0.13)). The significance of all results reported from the ANOVA remained the same, regardless of whether 56 or 45 participants were included in the analysis.
