# Supplementary figures and images for: Sleep restriction can attenuate prioritization benefits on declarative memory consolidation
Source: J Sleep Res. 2016 Jun 13;25(6):664–72. doi: 10.1111/jsr.12424 (PMC5324680; doi:10.1111/jsr.12424)

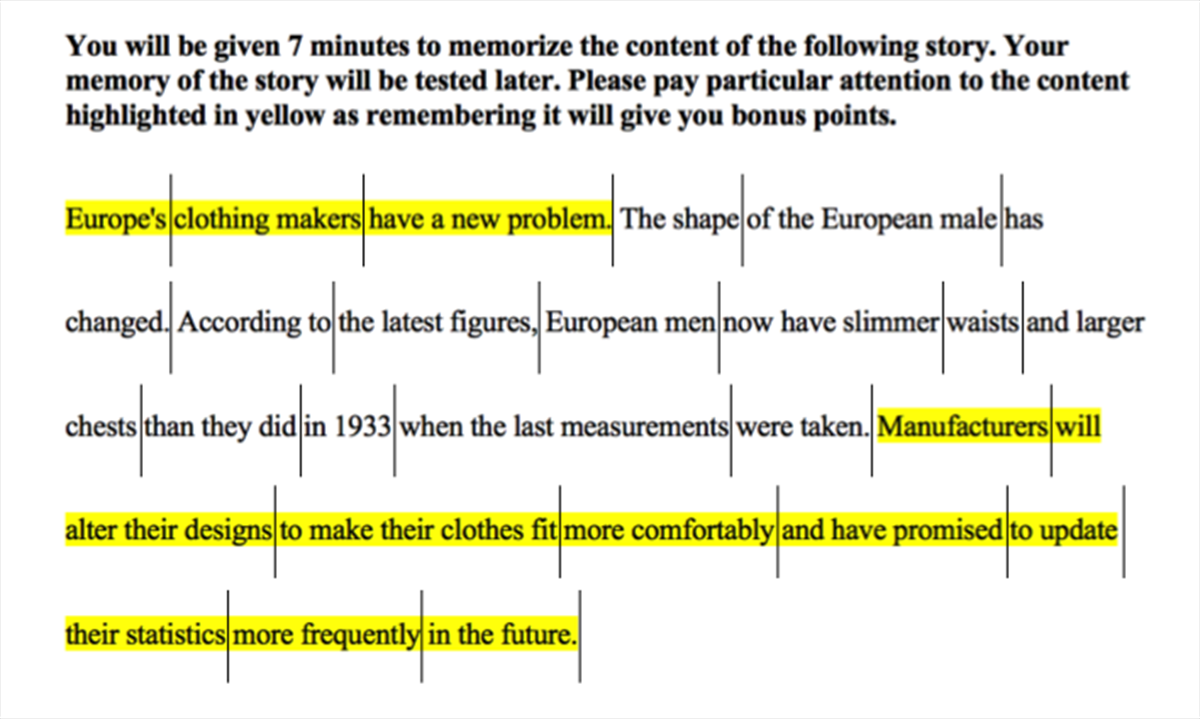

Supplement: Supplementary file 1 — Appendix S1. Information on participants' recruitment, screening, accommodations and daily activities during the 2‐week experimental protocol. For additional information, see Lo and colleagues (2016). [file JSR-25-664-s001.tif]

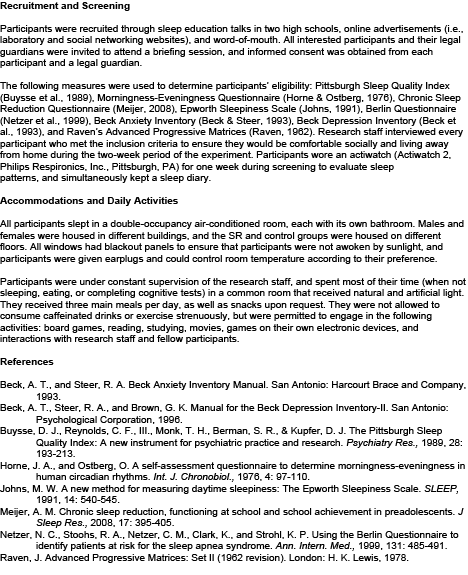

Supplement: Supplementary file 2 — Appendix S2. Prose passage read by participants, including instructions in bold type. The prioritization of content was manipulated by including highlighted and non‐highlighted statements, with participants told that they would receive an additional reward for subsequent memory of highlighted information. The vertical lines (shown here, but not to participants) depict the separation of idea units. [file JSR-25-664-s002.tif]

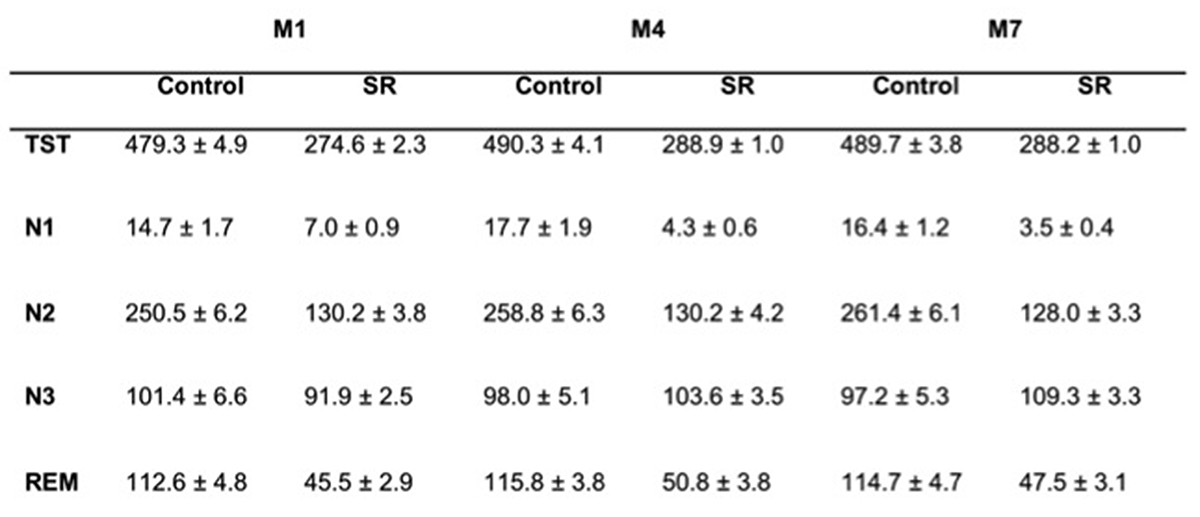

Supplement: Supplementary file 3 — Appendix S3. Sleep stage duration in minutes (mean ± standard error), separated by group (control, SR) and manipulation night (M1, M4, M7). [file JSR-25-664-s003.tif]
